# An Effective and Playful Way of Practicing Online Motor Proficiency in Preschool Children

**DOI:** 10.3390/children11010130

**Published:** 2024-01-20

**Authors:** Eleanna Adamopoulou, Konstantina Karatrantou, Ioannis Kaloudis, Charalampos Krommidas, Vassilis Gerodimos

**Affiliations:** Department of Physical Education and Sports Science, University of Thessaly, 42100 Trikala, Greecekokaratr@uth.gr (K.K.); hkrom@uth.gr (C.K.)

**Keywords:** children’s fairytale, distance learning, physical activity, kindergarten, motor skills, developmental ages

## Abstract

The children’s fairytale is a playful educational tool that can be modified in such a way to enhance motor proficiency. This study investigated the effect of an online exercise program with modified fairytales on children’s motor proficiency during the kindergarten curriculum. Forty preschool children (20 girls and 20 boys; 5.13 ± 0.24 years old) were divided into two equal groups: an intervention (IG) group and a control group (CG). The IG followed a 3-month (3 times/week) online exercise intervention program (supervised by the class’s kindergarten teacher) with modified children’s fairytales, during the COVID-19 pandemic, with an aim to improve their motor proficiency. The CG did not attend any exercise intervention program. The Democritos Movement Screening Tool for Preschool Children (DEMOST-PRE), consisting of ten tests, was used to assess the children’s motor proficiency. Τhe IG, after the end of the intervention program, significantly improved in the DEMOST-PRE total score (*p* < 0.001; mean change: 40.7%), while the CG did not significantly improve in the total score (*p* > 0.05). Furthermore, in the IG, a significant negative correlation between the pre-training DEMOST-PRE total score and the percentage change following the intervention (r = −0.64; *p* = 0.002) was observed. A modified exercise program using children’s fairytales may be incorporated into the kindergarten curriculum as an effective educational tool for the improvement of motor proficiency.

## 1. Introduction

Preschool age (from 3 to 6 years old) is an ideal period of a child’s life to improve motor proficiency [1,2], as well as to lay the foundations for lifelong physical activity and exercise [3,4]. According to Bruininks [5], “motor proficiency is determined by qualitatively different aspects of both gross and fine motor development and serves as an index of children’s motor development”. Children who have achieved motor proficiency levels identify themselves as being capable and socially accepted and they have positive attitudes towards physical activity and a healthy lifestyle in general [1,2,6]. On the other hand, preschool children with low levels of motor proficiency may experience lifelong motor skill problems as well as low levels of physical activity [1,2,6]. Although the importance of motor proficiency has been established worldwide, its level has been reported to be low in children [7]. For this reason, several previous studies designed and implemented different intervention programs [8], demonstrating promising results in motor development [9,10,11,12,13,14,15,16,17,18,19].

Kindergarten is a perfect environment to promote physical activity and improve motor proficiency in children through different educational and experience-based learning activities. Fairytales—Children’s stories—have a central role in the kindergarten curriculum [20]. They not only captivate the imagination of children but also enhance their creativity, critical thinking, and reasoning skills [20]. Although fairytales are present as a significant proportion in children’s education, the traditional way in which they are used does not contribute to physical activity promotion and motor proficiency improvement. However, studies are reporting the relationship between movement and speech, indicating significant benefits not only in the cognitive and socio-emotional domains but also in the motor domain [21,22,23]. Vidoni et al. [22] state that a story can be modified in such a way as to include specific knowledge and skills by enhancing physical literacy. According to Almond and Whitehead [24], physical literacy is “a fundamental and valuable human capability that can be described as a disposition acquired by human individuals encompassing the motivation, confidence, physical competence, knowledge and understanding that establishes purposeful physical pursuits as an integral part of their lifestyle”.

Lately, few studies have designed protocols and examined the effectiveness of combined movement and storytelling interventions in motor, cognitive, and/or linguistic skills in preschool children [25,26,27,28]. More specifically, Duncan et al. [25] reported that a 6-week (2 times/week; 30 min/time) combined movement and storytelling intervention program, in kindergarten, enhanced motor competence and language ability in preschool children to a greater extent than movement or storytelling alone. Furthermore, Eyre et al. [26] found that a 12-week combined movement and storytelling intervention program (1 time/week; 35 min/time), in kindergarten, significantly improved different motor skills in both White and South Asian preschool children. More recently, Vargas-Vitoria et al. [28] developed and designed a 12-week (3 times/week; 40 min/time) combined movement and storytelling intervention program for preschool children. The effectiveness of the above program on fundamental motor skills, language development, and physical activity level is expected to be examined by the authors in Chilean children.

It should be mentioned that all the aforementioned studies [25,26,28] designed and/or implemented combined movement and storytelling intervention programs in-person into kindergarten. While, there is no information regarding the effectiveness of an online (using live streaming distance learning) combined movement and storytelling intervention program, during the kindergarten curriculum, for preschool children. Lately, however, distance learning, has gained popularity (especially during the COVID-19 pandemic) in the educational community by offering flexibility in terms of time and place as well as reducing educational barriers due to geographic parameters [29,30], insufficient facilities, and equipment [31]. Previous studies mentioned that educators should follow and promote physical activity guidelines even during distance learning by incorporating movement-enhancing practices into their daily school-based online schedule [32].

Taking all the above into consideration, we designed the present study in an attempt to offer, in the scientific literature and the kindergarten educational community for the first time, an effective and playful way of practicing online motor proficiency. In more details, the main objective of this study was to investigate the efficacy of a 3-month (3 times/week) school-based online exercise program (using live streaming distance learning) with modified fairytales on preschool children’s motor proficiency. It was hypothesized that the intervention program with modified fairytales will improve motor proficiency in preschool children. It was also hypothesized that the baseline motor proficiency level will influence the percentage change following the intervention program. Thus, it was hypothesized that the participants with lower baseline levels in motor proficiency will report greater percentage change following the intervention program.

## 2. Materials and Methods

### 2.1. Participants

Forty preschool children (20 girls and 20 boys) aged 4–6 years old, with the signed consent of their parents, voluntarily attended the study. The children were divided into two equal groups: (a) the intervention group (IG; *n* = 20; 10 girls and 10 boys) and (b) the control group (CG; *n* = 20; 10 girls and 10 boys). The children were recruited from one kindergarten school, where the kindergarten teacher participated in the study and was responsible for the implementation of the measurements and the intervention. In this kindergarten, there were two classes with preschool children who participated in this study and each class was randomly assigned to either an IG or a CG. The age and the somatometric characteristics of the participants are presented in Table 1. Necessary conditions for the participation of children in the program were considered: (a) the assurance that the individual health card of each child had been submitted in the kindergarten records and (b) that the children were healthy and did not participate in any other form of organized physical activity during the three months. This was evaluated using a specific questionnaire from the American College of Sports Medicine [33].

### 2.2. Study Design

Before the start of the study, we received approval to conduct the study from the Ethics Committee of the University of Thessaly, as well as from the Institute of Educational Policy of Greece. Also, during the appointment for baseline measurements (live at the kindergarten), the parents were informed by the investigator regarding the online intervention procedure and their role during the program, and they signed an informed consent form. Then, the baseline measurements were performed on one day for each child. During the study, both the IG and CG continued to follow the daily curriculum of the kindergarten online via the Webex platform, because of the mandatory closure of schools due to the COVID-19 pandemic. All the online courses of the kindergarten curriculum in both groups (IG and CG) were led by the same kindergarten teacher. However, three days/week for 30 min/day (integrated into the daily curriculum), the IG performed the intervention of the present study with modified fairytales supervised by the kindergarten teacher. The CG did not participate in any physical activity intervention during the study, and it continued the daily online curriculum of the kindergarten without any differentiation. Two days after the completion of the 3 months, the pre-training measurements were repeated in the same order and at the same time of the day. All measurements (initial/final) were performed live at the kindergarten by the same investigator and lasted approximately 20 min per child. Although the kindergarten was closed due to the pandemic COVID-19, we requested and received special permission by the Directorate of Primary Education to carry out the initial and final measurements of the kindergarten students. Thus, each child with his/her guardian, observing all protection measures, was coming to the kindergarten after a prearranged appointment for their measurements. It should be mentioned that at all stages of the research, the health and safety instructions of the competent bodies for the protection of all those involved were implemented immediately and with special care. Finally, respecting children’s privacy, we did not take videos from the online interventions during the study.

### 2.3. Online Intervention Program with Modified Fairytales

The children of the IG participated from their own home in an online exercise program, during the kindergarten curriculum lasting three months, with a frequency of three times/week. The intervention program was performed through the Webex platform (live streaming using a camera) and supervised by the class’s kindergarten teacher. However, the parents had an important role in the intervention process by securing the equipment (according to each lesson) and shaping a free space that was necessary for the implementation of the lessons. A week before every lesson, the parents received a mailing list, from the kindergarten teacher, with the appropriate equipment.

Each training session lasted 30 min and included one modified children’s fairytale for the promotion of motor proficiency (36 fairytales have been used in total during the 3-month intervention program). Each modified fairytale consisted of different organized exercise activities for practicing (a) different motor skills such as locomotor skills (walking, running, jumping, limping, hopping, sliding, etc.), stabilization skills (stretching, rotation, turning, body rolling, balance, etc.), object control/manipulative skills (throwing, receiving, spinning, hitting, etc.), and (b) physical fitness parameters (flexibility, coordination abilities, speed, strength, and aerobic capacity). An indicative modified fairytale “Little Red Riding Hood” is analytically presented (the storyline, the integrated motor activities, the main objectives of the modified fairytale, etc.) in Table 2.

All the selected activities, the equipment, and the space used during the intervention program were based on the fact that the intervention program was performed using distance learning with each child in their own home. During the intervention period, special attendance books were filled in to confirm the participants’ adherence to the program. All the participants of the study, who were included in the statistical analysis, completed 36 sessions in total.

### 2.4. Testing Procedures

Before the start of the research, the basic anthropometric characteristics (body mass and body height) of the sample were evaluated using a physical beam scale (Seca model 220, Seca, Hamburg, Germany). Furthermore, before and immediately after the completion of the study, the children’s motor proficiency was tested with the DEMOST-PRE, as previously described by Kambas and Venetsanou [34,35]. During the testing procedure, both in pre- and post-training measurements, we used the fairytale procedure, as recommended by the developers. The DEMOST-PRE screening tool can be applied in any area of kindergarten, it is easy to use, and it has a short duration and a playful form as well as has high reliability (Cronbach a = 0.873) and validity (0.80) [34,35].

The DEMOST-PRE screening tool [34,35] includes ten tests:

(a) An introductory test (not evaluated) of the procedure (“hand preference”);
(b) Two tests of fine motor skills (“dots on paper/tapping” and “picking up and placing coins in an area”);
(c) Four tests of gross motor dexterity (“jumping repeatedly sideways”, “toe-to-heel walking backwards”, “stepping through 3 vertical hoops”, and “standing jump over a stick”);
(d) Two perceptual-motor tests (“overhead toss to a specific target” and “catching a bean bag”);
(e) A test of combined gross, perceptual-motor, and fine dexterity (“running and carrying and placing a ball in a box”).

The raw score of each task (e.g., time, number of dots, number of jumps) is recorded, and thereafter it is transformed to a point score [35]. Afterwards, those point scores are added to yield the total battery score (DEMOST-PRE total score) [35] that is used for the statistical analysis as proposed by the developers [35].

### 2.5. Statistical Analysis

A statistical power analysis (software package GPower 3.0), before the initiation of the study, indicated that a total number of 40 participants (20 participants in each group) would yield adequate power (>0.85) and a level of significance (<0.05). Data were analyzed with IBM SPSS Statistics v.26 software (IBM Corporation, Armonk, NY, USA) and the results are presented as means ± standard deviations. The normality of the distribution was examined using the Shapiro–Wilk test (all variables followed the normal distribution). Following that, the baseline measurements were examined if there were differences in the pre-intervention DEMOST-PRE total score between boys and girls using the *t*-test for independent groups. However, no statistically significant differences were found between boys and girls in pre-training measurements (*p* > 0.05). For this reason, the main statistical analysis of the study was performed and the results were presented using boys and girls as a whole sample. Thus, to examine the effect of the 3-month exercise intervention program on the improvement of DEMOST-PRE total score, a two-way ANOVA, “group” × “time” (2 × 2) was used, with repeated measurements on the “time” factor and multiple comparisons with the Sidak method where necessary. The magnitude of the difference (effect size) was assessed by Cohen’s d. The independent *t*-test was also used to examine possible differences in the percentage change (from pre- to post-measurement) between the two groups. Finally, the Pearson correlation test was used to examine a possible association between the pre-training DEMOST-PRE total score and the magnitude of percentage change (from pre- to post-measurement). The significance level was set at *p* < 0.05.

## 3. Results

**Figure 1:**
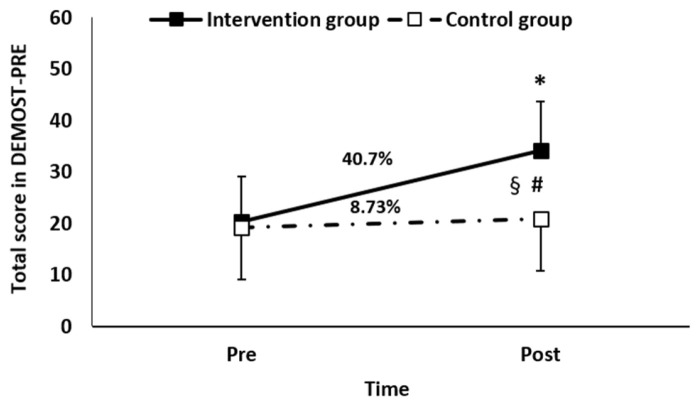
The DEMOST-PRE total score in the intervention and control groups pre- and post-training (mean ± standard deviation) where * *p* = 0.000 vs. pre-training in IG; # *p* = 0.000 vs. CG in the post-training measurement; § *p* = 0.000 vs. CG in the percentage change.

Repeated measures analysis of variance revealed a statistically significant “group” and “time” interaction effect in the total score of the DEMOST-PRE (*F*_1,38_ = 32.58; *p* = 0.000). In the IG, a statistically significant difference (increase in the total score of the DEMOST-PRE) was observed between the initial (20.28 ± 8.89 score) and final (34.21 ± 9.5 score) measurements (*p* = 0.000; *d* = 1.51 large effect size; Figure 1). In the CG, the total score of the DEMOST-PRE remained stable (*p* = 0.139; pre = 19.20 ± 10 score and post = 20.84 ± 9.58 score). Regarding the differences between the groups, the initial measurement did not differ significantly (*p* = 0.481) in contrast to the final measurement where a statistically significant difference was observed between the two groups, with the IG being superior to the CG (*p* = 0.001; *d* = 1.40 large effect size; Figure 1). Furthermore, the independent *t*-test showed a significant difference in the percentage change (from pre- to post-measurement) of the total score between the two groups (*t*_(38)_ = 3.91; *p* = 0.000; Figure 1).

The raw and point scores at each task as well as the DEMOST-PRE total score per group (intervention and control group) and measurement (pre- and post-training) are analytically presented (mean ± standard deviation) in Table 3.

**Figure 2:**
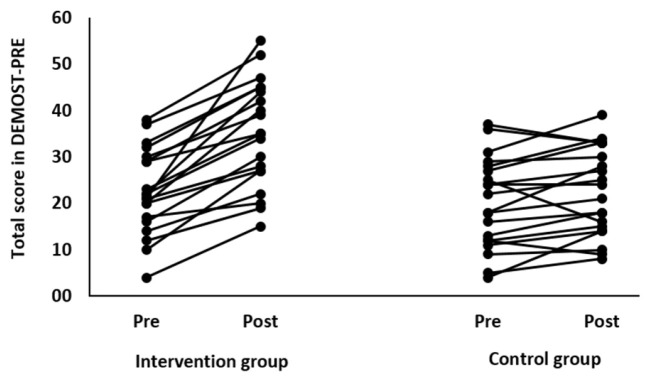
Individualized change (per participant) from pre- to post-training in the intervention and control group.

When the results were analyzed and presented individually, we found that all the participants of the IG, following the intervention program, improved in the DEMOST-PRE total score. Although, as we mentioned earlier, the mean percentage change in the DEMOST-PRE total score was 40.7%; this ranged from +15% to +73.33% depending on the participant. Regarding the CG, when the results were examined individually, we observed conflicting results. Analytically, in some children in the CG, the DEMOST-PRE total score remained stable, while in others this either increased or decreased following the 3 months. The individualized changes from pre- to post-training in the IG and CG are presented in Figure 2.

**Figure 3:**
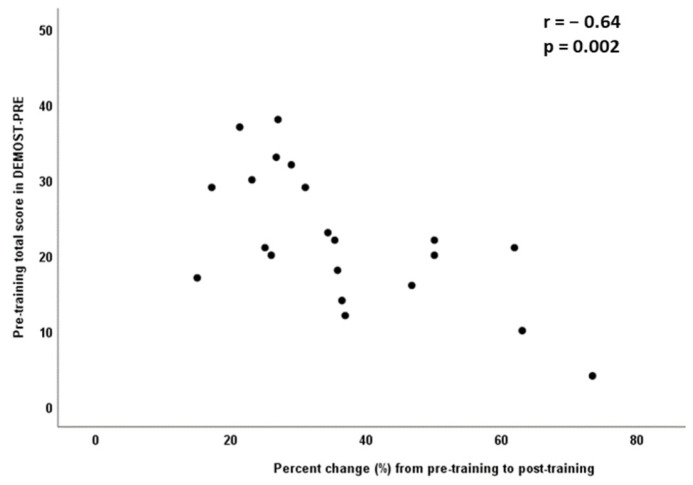
Relationship of pre-training DEMOST-PRE total score with the percentage change following the 3 months in the intervention group.

Finally, in the IG, Pearson correlation analysis revealed a significant negative correlation between the pre-training DEMOST-PRE total score and the percentage change following the intervention program (r = −0.64; *p* = 0.002). The participants with lower pre-training levels in DEMOST-PRE total score reported greater percentage change following the intervention program. The relationship of the pre-training DEMOST-PRE total score with the percentage change following the intervention program is depicted in Figure 3.

## 4. Discussion

The present study designed, implemented, and evaluated the efficacy of an online (live streaming distance learning) physical activity program with modified children’s fairytales, during the kindergarten curriculum, on improving motor proficiency. Our results demonstrated that a 3-month (3 days/week) online physical activity program with modified children’s fairytales, incorporated into the kindergarten curriculum, induced significant improvements in the children’s motor proficiency (mean percentage change: 40.7%); as this was evaluated using the DEMOST-PRE battery score.

As we mentioned earlier, this is the first study that examined the efficacy of an online combined movement and storytelling intervention program on the motor proficiency of preschool children. To date, the few studies that examined the effect of combined movement and storytelling intervention programs in preschool children have been performed inside the kindergarten school and those studies seem to agree with the results of the present study. In more detail, our findings are in line with the findings of Eyre et al. [26], who indicated significant improvement in motor proficiency through a 12-week combined (1 time/week; 35 min/time) movement and storytelling intervention program in kindergarten in both White and South Asian preschool children. Following the intervention, all children improved their motor skills, with a bigger improvement observed for South Asian (mean change: 32.96%) compared to White (mean change: 11.3%) children [26]. In our study, the mean percentage change in the DEMOST-PRE total score was 40.7% (range of change: +15% to +73.33%), which greater than that observed in South Asian and White children [26]. The greater improvement observed in the present study compared to that of Eyre et al. [26] may be attributed to the frequency of the intervention program (3 times/week in the present study vs. 1 time/week in the Eyre et al. study). It should be also noted that in our study, as well as in the study of Eyre et al. [26], significant association has been demonstrated between the baseline motor skill levels and the magnitude of improvement. More specifically, children with lower baseline motor skill levels showed greater improvement following the intervention program.

Furthermore, our results follow that of Duncan et al. [25], who reported that a 6-week (2 times/week; 30 min/time) combined movement and storytelling intervention program, in kindergarten, improved motor competence in preschool children. In the above study [25], motor competence significantly improved following the intervention; however, the magnitude of change was significantly greater for the combined movement and storytelling group (Δ = 4.86) compared to the movement-only (Δ = 2.88) or storytelling-only (Δ = 1.77) groups. In the same context, Rezaei et al. [27] reported that a 12-week (2 times/week; 35 min/time) combined movement and storytelling intervention program improved motor skills to a greater extent than movement or storytelling alone in children with developmental coordination disorders. The use of a combined movement and cognitive intervention approach (i.e., a language-focused storytelling intervention) may induce additional benefits in both movement and cognitive domains either as a result of embodied cognition via sensorimotor experience or enhanced blood flow to the brain with higher intensities of physical activity [25].

The results of the present study are also in line with those of other researchers, who designed and implemented different traditional playful physical activity or general motor skill programs and who reported significant improvement in the motor proficiency of preschool children [9,10,11,12,13,14,15,16,17,18]. All the above programs for the practice of motor skills in the aforementioned studies have as a common denominator the involvement of children in a wide range of motor activities promoting physical literacy. Wick et al. [36] mentioned that motor skills need to be taught, practiced, and supported repeatedly to be improved and maintained throughout life. The absence or the interruption of physical activity may affect the increase in motor skills during the developmental years. This notion could be strengthened by the results of the present study where the control group (without participating in any organized physical activity program during the 3 months) did not significantly change motor proficiency. In the control group, the absence of motor proficiency improvement may be attributed to the COVID-19 pandemic situation, where in Greece the confinement and the absence of physical activity (closed schools, closed sports clubs, etc.) may have negatively influenced the physical fitness levels and impeded the development of motor skills in children. This notion is supported by several previous investigators from different countries who reported impaired physical fitness and motor skill levels in children during the COVID-19 pandemic [37,38,39].

Furthermore, the findings of the study revealed that there was no statistically significant difference in motor proficiency between the two sexes in pre-training measurements. The results of the present study strengthen the findings of previous studies that show no differences in motor performance between preschool boys and girls [16,40,41]. These results are also supported by Gallahue and Ozmun [42], who mention that the physical characteristics of preschool children (3–5 years old) are the same for both genders. Gallahue [2] states that each child has a unique timing and pattern of growth and development that is not affected by gender. On the other hand, a previous study [40] showed that girls outperformed boys in rhythmic ability tests, suggesting the incorporation of specific rhythmic activities (like Orff, Dalcroze, and dance) into the kindergarten curriculum to overcome the boys’ performance deficiency.

The present study has some limitations that could affect the generalization of its outcomes. The findings of this study are limited to the use of a 3-month online (live streaming distance learning using the Webex platform) physical activity program for motor proficiency development using modified fairytales, where each child was in their own home. Future studies could investigate and compare the efficacy of analogous programs in-person inside the kindergarten. Furthermore, our findings are clearly limited to healthy preschool boys and girls; future studies should compare the efficacy and safety of equivalent programs in preschool children with health problems or in children of other age groups. Also, our study is limited to the use of an intervention with modified fairytales without comparing its efficacy with other interventions. Future studies could compare similar interventions with modified fairytales with other more typical or traditional interventions for motor proficiency development in preschool children. Finally, our findings are limited to the evaluation of motor proficiency, while no other parameters were evaluated. A future study, in addition to motor skills, could evaluate for example children’s cognitive abilities, due to the use of verbal storytelling in the intervention.

## 5. Conclusions

The results of the present study have important practical implications, demonstrating that a 3-month (3 times/week) online exercise program with modified fairytales, incorporated into the kindergarten curriculum, may lead to significant improvements in children’s motor proficiency. The physical activity program of this study, using modified fairytales, may safely be used by kindergarten teachers as an efficient and playful way for the promotion of motor proficiency in preschool children. However, in order to secure the safety of the children and the efficacy of the program, the kindergarten teacher should have received appropriate education on this topic. Additionally, when the intervention is performed online (as in the present study), it is important to ensure (a) the safe implementation and guidance of children in the program under the continuous online supervision of a teacher (using the live streaming method) and (b) the collaboration of parents. If these two factors are not controlled, it may negatively influence the safety and efficacy of the online program.

## Figures and Tables

**Table 1:** Age and somatometric characteristics of the participants (mean ± standard deviation).

Characteristics	IG (*n* = 20)	CG (*n* = 20)
Age (years old)	5.10 ± 0.20	5.16 ± 0.28
Body mass (kg)	22.81 ± 4.16	21.87 ± 4.16
Body height (m)	1.15 ± 0.05	1.14 ± 0.03
BMI (kg/m^2^) *	16.93 ± 2.36	16.59 ± 2.48

* BMI: body mass index = body mass/body height^2^; IG: intervention group; CG: control group.

**Table 2:** Modified fairytale: “Little Red Riding Hood”.

Objectives: 1. Motor skills (object control, locomotor, and stabilization skills), 2. physical fitness (flexibility, coordination abilities, speed, strength, and aerobic capacity), 3. cognitive domain (body parts and animals), and 4. emotional domain (perceived ability). Total duration: ~30 min (6 min story narration and 24 min exercise activities). Training contents: 7 exercise activities. Training equipment: 10 small toys, 2 large bags, 4 books, 3 water bottles, paper tape.	
Story	Transition–Exercise activities
Once upon a time, a little girl lived with her parents in the forest. Her father was a lumberjack and all day he worked in the forest cutting wood, making bundles, and selling them.	Transition: At this point, let us help her dad to gather more wood. Exercise activity 1: We invite the children to move the 10 bundles (“small toys”) from one side to the other (the children place the “small toys” in two large bags). The activity is repeated 3 times (with 40 s rest between repetitions).
One day the grandmother, who loved the little girl very much, gave her a red coat with a red hood. Can you imagine what the name of the little girl is? … So the “Little Red Riding Hood” had a great time at home. Every day she went out in her yard and started playing with her toys.	Transition: Let us play with her. Exercise activity 2: We invite the children to run quickly and freely in the area we have demarcated (3 m × 6 m). Each time we mention a body part, the children must stop and touch with their hand the mentioned body part (arm, leg, knee, back, elbow). The activity is repeated 5 times (5 times × 10 s running, with 5 s pause between repetitions to touch the called body part).
Suddenly, one day while she was playing, her mom called her and told her: “Grandma is very sick, you have to give her food to make her stronger. Be careful on the road and do not leave the path because you will get lost. Don’t forget to stay away from the Wolf!!” Little Red Riding Hood starts off happily on her big walk in the forest.	Transition: As she walked in the forest, she noticed that there were many obstacles in the path. She had to cross them to continue her way and not get lost. Exercise activity 3: We design, in the room, one track with “obstacles” and the children are called to cross them (jump with both feet forward two “books” in a row, balance on a route of 3 m paper tape, and zig-zag between 3 bottles). The activity is repeated 3 times with 30 s rest between the repetitions.
After she managed to cross the obstacles, she continued happily, BUT suddenly she met the WOLF in front of her.	Transition: Trying to hide from him… Exercise activity 4: The children move by crawling to the point we have set (3 m distance with paper tape). The activity is repeated twice with 40 s rest.
In the end, she managed to cross its path. After she continued on her way, she finally reached her grandmother’s house. However, the wolf tricked her, scared her, and suddenly appeared in front of her. - Where are you going? the wolf asked her. - I am going to give my grandmother some food because she is sick, Little Red Riding Hood answered him. - And where does your grandmother live? The wolf asked. - It’s not your business dear wolf… Little Red Riding Hood answered.	Transition: And then Little Red Riding Hood started running–running–running until she managed to get away from the wolf. Exercise activity 5: Children begin to run freely in the space we have demarcated (3 m × 6 m) by lowering their center of gravity and taking quick and gentle steps (duration 3 min).
However, the wolf did not give up. He was hungry, VERY hungry. He knew that there was only one house at the end of the path, so he thought that Little Red Riding Hood would probably go there. So, he decided to hide behind a tree and wait to see. In the meantime, Little Red Riding Hood came out of her hiding place and continued her way until she reached outside her grandmother’s house.	Transition: Little Red Riding Hood, instead of going inside, decided to play with the animals she met around the house. Exercise activity 6: The children start imitating the walk of the animals that we mentioned that Little Red Riding Hood met (elephant, frog, eagle) in the space (3 m × 6 m) we have demarcated (duration 5 min).
So, the wolf took the opportunity and entered the grandmother’s house first. TOK TOK TOK, he knocked on the door. - Who is there? Grandma asked - I am Little Red Riding Hood, answered the wolf, changing his voice. Please open for me to enter. I bring you fresh food. - Oh, come in, my little girl, said the grandmother. Suddenly when the grandmother opened the door, the wolf ate her and then he lay down comfortably on the bed waiting for Little Red Riding Hood to arrive… Little Red Riding Hood, after finishing her game with the animals in the yard, entered her grandmother’s house and she went to give her the food basket. Then the wolf got up and he ate Little Red Riding Hood as well. Later in the evening, a hunter was passing, he saw the door of the grandmother’s house open, and he was surprised. He entered the house and he saw the wolf sleeping. He looked more carefully and saw that something was moving in his tummy. Therefore, he decided to open the wolf’s tummy. SUDDENLY, the grandmother and Little Red Riding Hood came out. - WOW! How scared am I, said Little Red Riding Hood and gave her grandmother a big hug.	Transition: For so long in the wolf’s belly, we were caught. Let us stretch with Little Red Riding Hood and her grandmother. Exercise activity 7: We stretch our arms high and try to reach the sky and we fold our body trying to reach our toes, inhaling through the nose exhale through the mouth (3 sets × 8 reps, 30 s rest between sets).

**Table 3:** Raw and point scores at each task as well as the DEMOST-PRE total score in the two groups pre- and post-training (mean ± standard deviation).

Testing Variables	Group	Pre-Training	Post-Training
		Raw Scores	Raw Scores
Dots on paper/tapping (number of dots)	IG	31.40 ± 13.04	42.45 ± 14.09
	CG	29.55 ± 12.67	31.30 ± 12.81
Picking up and placing coins in an area (time in s)	IG	50.20 ± 10.50	46.5. ± 12.50
	CG	50.10 ± 11.40	49.60 ± 11.50
Jumping repeatedly sideways (number of succesful jumps)	IG	3.10 ± 2.19	5.10 ± 2.30
	CG	2.74 ± 2.40	2.63 ± 2.48
Toe-to-heel walking backwards (number of succesful steps)	IG	5.38 ± 5.08	10.57 ± 5.24
	CG	4.32 ± 4.87	5.68 ± 5.61
Stepping through three vertical hoops (number of succesful trials)	IG	0.71 ± 0.78	0.95 ± 0.66
	CG	0.74 ± 0.61	0.73 ± 0.58
Standing jump over a stick (number of succesful jumps)	IG	2.33 ± 1.57	2.76 ± 1.20
	CG	2.58 ± 1.40	2.47 ± 1.28
Overhead toss to a specific target (number of succesful tosses)	IG	3.19 ± 2.29	4.76 ± 2.59
	CG	3.00 ± 2.45	2.95 ± 1.96
Catching a bean bag (number of succesful catches)	IG	1.14 ± 1.24	1.38 ± 1.20
	CG	1.12 ± 1.09	1.14 ± 1.19
Running and carrying and placing a ball in a box (time in s)	IG	16.39 ± 3.65	13.86 ± 1.72
	CG	15.99 ± 2.21	15.22 ± 2.00
**Transformation**			
**Testing Variables**	**Group**	**Pre-Training**	**Post-Training**
		**Point Scores**	**Point Scores**
Dots on paper/tapping	IG	1.19 ± 1.69	2.76 ± 3.14
	CG	0.84 ± 1.57	1.26 ± 2.08
Picking up and placing coins in an area	IG	2.00 ± 0.5	3.00 ± 0.40
	CG	2.00 ± 0.6	2.10 ± 0.5
Jumping repeatedly sideways	IG	3.10 ± 2.19	5.10 ± 2.30
	CG	2.74 ± 2.40	2.63 ± 2.48
Toe-to-heel walking backwards	IG	5.38 ± 5.08	10.57 ± 5.24
	CG	4.32 ± 4.87	5.68 ± 5.61
Stepping through three vertical hoops	IG	0.71 ± 0.78	0.95 ± 0.66
	CG	0.74 ± 0.61	0.73 ± 0.58
Standing jump over a stick	IG	2.33 ± 1.57	2.76 ± 1.20
	CG	2.58 ± 1.40	2.47 ± 1.28
Overhead toss to a specific target	IG	3.19 ± 2.29	4.76 ± 2.59
	CG	3.00 ± 2.45	2.95 ± 1.96
Catching a bean bag	IG	1.14 ± 1.24	1.38 ± 1.20
	CG	1.12 ± 1.09	1.14 ± 1.19
Running and carrying and placing a ball in a box	IG	1.24 ± 1.22	2.43 ± 1.25
	CG	1.68 ± 1.16	1.88 ± 1.41
DEMOST-PRE total score	IG	20.28 ± 8.89	34.21 ± 9.5
	**CG**	**19.20** **± 10**	**20.84 ± 9.58**

## Data Availability

The data presented in this study are available on request from the corresponding author. The data are not publicly available due to privacy or ethical restrictions.
